# Examining General Vaccine Acceptance and COVID-19 Vaccine Intention: Comparison across Pharmacies in California and Ohio

**DOI:** 10.3390/pharmacy11020046

**Published:** 2023-02-27

**Authors:** Amanda R. Mercadante, Aleda M. H. Chen, Vivian Chu, Jason C. Wong, Anandi V. Law

**Affiliations:** 1 College of Pharmacy, Western University of Health Sciences, 309 E 2nd St, Pomona, CA 91766, USA; 2 School of Pharmacy, Cedarville University, 251 N. Main St., Cedarville, OH 45314, USA

**Keywords:** vaccine hesitancy, vaccine acceptance, COVID-19, community pharmacy

## Abstract

Given the complexities surrounding vaccine acceptance of COVID-19 and other vaccines, it is important to determine the underlying health beliefs of patients in order to bridge gaps and promote vaccine confidence. With pharmacies as key hubs for vaccinations and vaccine conversations, examining patient perspectives through the lens of community pharmacy may provide a targeted insight into their patient populations. The primary objectives of this study were to measure COVID-19 vaccine intention and compare vaccine acceptance at pharmacies and clinics between California and Ohio. The secondary objectives included subgroup comparisons of vaccine intention and vaccine acceptance based on demographic characteristics. A previously validated survey instrument (5C survey tool) was administered at pharmacy sites in California and Ohio to examine respondents’ vaccine acceptance (confidence, complacency, constrains, calculation, and collective responsibility). Additional items were added to capture flu and COVID-19 vaccine intention. Reliability and confirmatory factor analysis were completed for the 13-item 5C. Comparisons were made between sites and within different demographic groups. Good reliability (Cronbach’s alpha = 0.768) was found, with nearly all items loading on their hypothesized domains. Respondents from Ohio had significantly higher complacency and constraints domain scores. Highest acceptance was revealed in females, individuals with a Master’s degree or higher, and individuals with the intention to receive a flu vaccine. The adapted 5C is a reasonable tool to measure vaccine intention in English-speaking populations in the US. Certain demographic groups may have lower vaccine acceptance; pharmacists could consider implementing a tool, such as the 5C tool, to identify low acceptance. Given that the 5C tool gathers information on different domains of vaccine acceptance, healthcare professionals could utilize these results to improve trust and vaccine confidence in their patient populations; focused conversations concerning any of the respective domains could best address individual concerns and barriers about vaccinations, notably the COVID-19 and flu vaccines.

## 1. Introduction

SARS-CoV-2 (COVID-19) has had significant mortality and morbidity worldwide, with nearly 644 million cases and 6.6 million deaths as of 12 December 2022 [1]. From the early stages of the pandemic, the world anticipated the advent of vaccines that would prevent mortality as well as the transmission of the virus. With the approval of several vaccines beginning in late 2020 and early 2021, vaccines with high efficacy rates in preventing COVID-19 and mortality have become fully available for individuals aged six months and older [2]. Yet, as of April 2022, only approximately 65.8% of US adults have been fully vaccinated against COVID-19, with 82% receiving at least one dose of a vaccine. In December 2022, 13% of the population aged five and older were vaccinated with an updated booster dose [3].

Vaccine hesitancy, defined as “delaying in accepting or refusing vaccination despite availability of a vaccine” [4], has previously been listed by the World Health Organization as one of the 10 greatest threats to global health in 2019 [5]. Hesitancy can occur for a variety of reasons and contributes to lower than desired vaccination rates for many diseases or illness, including influenza [6], and of more current necessity, COVID-19 [3]. While it is often related to confidence in a vaccine, individuals may have deeper reasons for hesitancy. Betsch and colleagues (2018) posited the “5C psychological antecedents of vaccination”. They found the five key elements that explain much of the behavior surrounding vaccination: confidence (attitudes/beliefs), complacency (risk perceptions), constraints (barriers), calculation (the amount of information searching undergone), and collective responsibility (views on protecting others) [7]. Thus, this “5C” model may provide a valuable framework to help further define vaccine hesitancy for healthcare providers and assist them in promoting vaccine acceptance.

Various factors such as the vaccines’ “rapid” development, projected efficacy and safety, individual trust in healthcare and government systems, and health equity may contribute to some reported lack of confidence in the available COVID-19 vaccines [8]. Additionally, biased media sources, changing guidelines among states, and misinformation have affected patients’ understanding of COVID-19 and the available vaccines [9]. With influenza, patient health beliefs (more specifically as applied to the Health Belief Model), drove patient intent to vaccinate [10]. This may not be the same with COVID-19; Mercadante and Law (2018) found that during the early COVID-19 pandemic period, changes (general health habits, perspective/attitude, public awareness, and mental health) caused by COVID-19 did not influence a patient’s intention to vaccinate [11]. While it is important for healthcare professionals to understand the “why” behind a patient’s acceptance or refusal to receive the COVID-19 vaccine and build confidence [8], understanding the rates of hesitancy and acceptance and the underlying health beliefs may be of equal importance to bridge gaps between vaccine acceptance and patients, as well as promote more effective conversations about vaccines. Malik and colleagues revealed that there were notable differences in geographic disparities in vaccine acceptance in the United States; however, this study did not utilize a previously validated vaccine acceptance tool to obtain these results [12].

By understanding the differential impact of COVID-19 on the health and life of patients, and whether it differs by geographic area using the “5C model”, key evidence could be generated to better identify patients who are at greater risk for lower vaccine acceptance, and allow for more targeted conversations to improve vaccine confidence. Given the prominent role that pharmacists have taken to combat COVID-19 and influenza through vaccinations, in addition to the continued utilization of community pharmacies as immunization centers for key vaccinations, examining patient perspectives through the lens of community pharmacy may provide a targeted insight into their patient populations.

The primary objectives of this study were to measure COVID-19 vaccine intention and compare vaccine acceptance (using the 5C model of the antecedents of vaccination to determine the likelihood of acceptance) at pharmacy and clinical sites between California and Ohio. Secondary objectives included subgroup comparisons of vaccine intention and vaccine acceptance based on demographic characteristics.

## 2. Materials and Methods

This study included a prospective survey design at several different sites in California and Ohio that employed pharmacists who administered vaccines. Utilizing multiple sites allowed for extrapolation to urban and rural areas and different patient backgrounds. A survey using an electronic platform (Qualtrics) was designed and implemented to further test an instrument measuring the 5C model of the psychological antecedents of vaccination [7] and further examine vaccination hesitancy. These outcomes expanded on prior work by Mercadante and Law [11] by exploring regional data more deeply. The Consensus-Based Checklist for Reporting of Survey Studies (CROSS) was followed for this study [13]. This study was approved as exempt by the Western University of Health Sciences Institutional Review Board.

### 2.1. Outcomes Measures

Participants were directed to consider certain vaccines when answering items. Study outcomes included scores from the 5C survey and vaccination intention items. Demographic information was also collected for group comparison and to ensure a representative sample.

The 5C survey utilized contained the 4-domain, 13-item adapted version from the authors’ previous work [11]. In brief, the four domains were: confidence/collective responsibility, constraints, calculation, and complacency. Additionally, the authors included items to address vaccine intention, and demographics to identify which characteristics were associated with intention and acceptance. The work of Betsch and colleagues informed the integration of the “5C psychological antecedents of vaccination” [7]. The 5C was scored on a Likert Scale of Agreement (Strongly Disagree = 1, Disagree = 2, Agree = 3, and Strongly Agree = 4) with a potential score range of 13 (minimum, lowest likelihood of vaccine acceptance) to 52 (maximum, highest likelihood of vaccine acceptance). Face and content validity were performed on the instrument, as outlined in prior work; in sum, the instrument was assessed by four lay individuals and four healthcare professionals [11]. This instrument was designed to look at vaccines overall and not be vaccine-specific. Directions could be added to encourage the respondent to focus on a specific vaccine; however, at the time of the study, there were less specific concerns surrounding COVID-19 vaccines.

The second element of the survey contained patient-reported data regarding vaccination intention. The vaccination intention items were included to provide self-reported interest in receiving the COVID-19 vaccine at the time of the survey administration. Some of the surveys were administered prior to the advertisement, availability, and U.S. Food and Drug Administration (FDA) approval of the Pfizer and Moderna vaccines in December 2020, while some surveys were administered after the FDA emergency use authorization. However, at the time of administration, all respondents were aware of a vaccine nearing availability. Due to the concurrent flu season and concerns regarding a potential “twindemic” [14], additional intention items allowed the respondent to report if they received or planned to receive the flu shot as well as request their previous year’s vaccination uptake for the flu.

The third portion of the survey gathered demographic information including the following variables: number of prescription medications, gender identity, age group, race/ethnicity, highest educational qualification, and household income. To address the role of the perceived impact of COVID-19 on vaccination behavior, which was identified as a potential factor in prior work [11], an additional item included reported knowledge on whether the respondent knew someone who was impacted by the COVID-19 pandemic.

### 2.2. Recruitment, Sampling, and Data Collection

Sites were university-affiliated and were chosen out of convenience due to research connections. The Ohio recruitment site was at a pharmacy that is located in a small village (population: 4075) approximately 30 min from Dayton, Ohio (population: 140,569), which provides services to rural and urban patients within its area as well as college students in the community. It is also connected to a local School of Pharmacy and serves as a faculty, fellowship, and student experiential site. In California, two university-affiliated sites were utilized, including a federally qualified health center (FQHC) and independent pharmacy within Los Angeles County, which are urban settings. The independent pharmacy site in California was a pharmacy located in the city of Rosemead (population: 53,764), approximately 14 miles east of downtown Los Angeles. Rosemead is part of west San Gabriel Valley with growing Asian and Latino communities. Asian residents accounted for 48.6% of Rosemead’s population. The pharmacy is part of a local pharmacy franchise specializing in caring for Medicare and Medi-Cal patients and is affiliated with the authors’ (Western University of Health Sciences) College of Pharmacy as a faculty and student experiential site. The FQHC is located in San Gabriel Valley with eight health centers. It provides healthcare services to most major insurances and provides discounted healthcare via a sliding fee discount for uninsured patients. It is also affiliated with Western University of Health Sciences and serves as an experiential site for faculty, residents, and students.

The Inclusion criteria for respondents were the following: ≥18 years of age, able to independently complete survey questions, and able to understand one of the four languages available for the survey (English, Vietnamese, Traditional Chinese, or Spanish). Respondents provided consent to participate as part of the survey instructions and were terminated immediately before the survey began if they did not agree.

The desired sample size for comparative analyses were ≥30 respondents from each site, assuming a moderate effect size for the ANOVA analyses. Using Raosoft, to achieve a confidence level of 90% and a margin of error of 5% for the surveys, unknown population size, and response distribution of 50%, the minimum recommended total sample size was 267. While the minimums for each site in California were not achieved, they were achieved in Ohio. The total sample size was greater than 267.

Data were collected via paper surveys (Ohio) or Qualtrics (California) from 21 October 2020 to 30 March 2021. The participating sites were able to choose their preferred data collection method. Respondents could complete the surveys in their preferred language, if available, as noted above. Patients who entered the pharmacy were asked to complete the survey. A trained research assistant in Ohio performed data entry into Qualtrics from the paper surveys and the authors compiled all results into SPSS 26.0 for analysis.

### 2.3. Statistical Analyses

Analyses were conducted in alignment with the previous panel study by authors (ARM and AVL). There were no possibilities for missing data per the design of the survey and required responses. Descriptive statistics were completed for demographic and vaccination intention items. One-sample scale reliability of the adapted 13-item 5C was tested and measured by Cronbach’s Coefficient alpha. Construct validity was tested through factor analysis with Varimax rotation with an expectation of four resulting domains. Convergent and divergent validity were examined using item-item and inter-domain correlations from the confirmatory factor analysis.

To test the primary objective, correlational analyses were conducted between the 5C scores and vaccination intention items. An independent *t*-test was used to compare scores between CA and OH, and Levene’s test for equality of variances was completed where required. Analysis of Variance (ANOVA) was used to determine any differences between groups within CA and OH and their 5C scores (both individual domain scores and total 5C score). Tukey’s honestly significant difference (HSD) test was completed where required.

## 3. Results

A total of 355 surveys were completed and eligible for data analysis. Table 1 exhibits respondent demographics and responses to vaccine intention items. There were significant differences between Ohio and California respondents. Notable differences included how Ohio’s predominantly 18–29 year old white, college-educated sample has a generally higher income than those in the California group.

### 3.1. Reliability and Validity Evidence

Construct validity for the 13-item 5C instrument can be found in Table 2. The 13-item 5C showed good reliability with Cronbach’s alpha = 0.768. Confirmatory four-factor analysis with Varimax rotation revealed nearly all items loaded on their previously hypothesized domain. The exception was “Vaccine-preventable diseases are not so severe that I should get vaccinated”. While it loaded weakly across the four components, it was calculated into the Complacency domain when determining respondent scores. Item-item loading in all other domains was strong, ranging from 0.648 to 0.856.

### 3.2. Vaccine Intention, Complacency, and Acceptance

Scores for the domains of the 5C instrument were summed up and compared between Ohio and California (Table 3). Respondents from Ohio had significantly higher complacency and constraints domain scores. No other differences between the groups were significant. Table 4 presents the associations between the 5C domains and demographic characteristics as well as vaccine intention. There were significant differences in the total 5C score between different gender identities, with females having the highest scores. Hispanic/Latinx respondents had significantly higher complacency domain scores. Respondents with a Master’s degree or higher had significantly higher confidence/collective responsibility domain scores (vs. some college), complacency domain scores (vs. some high school and high school diploma), constraints domain scores (vs. high school diploma), and total scores (vs. some high school, high school diploma, and some college). Additionally, there were differences in other domains, as noted in Table 4. Regarding prescription medications, patients who reported taking 3–4 medications regularly had significantly higher confidence/collective responsibility domain scores than those with zero medications.

Respondents who reported receiving the flu vaccine last year (2019), had already received the flu vaccine this year (late 2020–early 2021), or planned on receiving it this year, had significantly higher scores in most domains as well as in the vaccine acceptance total scores. Respondents who reported having someone close to them who had been significantly impacted by the pandemic had higher complacency domain scores than those who did not. Respondents who indicated that they planned on getting the COVID-19 vaccine immediately when available had significantly higher scores than those who did not plan on getting the vaccine due to side effects or because of a lack of perceived need for most domains and for total scores.

## 4. Discussion

Respondents from Ohio had significantly higher domain scores of complacency and constraints. Highest acceptance was revealed in females, individuals with a Master’s degree or higher, and individuals with the intention to get a flu vaccine. Validity evidence verified the utility of the instrument, with all items but one loading on the hypothesized domains and strong item-item correlations in most domains. Previous work examining this instrument also demonstrated moderately strong reliability in its initial iteration [12], with stronger reliability in a subsequent iteration and strong construct validity from factor analysis [15]. Thus, the adapted 5C is a reasonable tool to measure vaccine intention in the US, as we were able to capture COVID-19 vaccine intention and factors surrounding the likelihood of receiving the vaccination, whether when immediately available or at specified time periods post-availability.

It is important to note that the 5C instrument is a general measure of vaccine intention, not COVID-19-specific, which increases the utility across a broad variety of vaccines. The authors did not alter questions within the 5C to fit COVID-19. Instead, the motivations and goal of the survey were explained through the instructions and addition of COVID-19 specific items to the survey (e.g., pandemic impact on others and intention to vaccination) for added correlational analyses or providing direction to the participants to consider certain vaccines when answering items. Other researchers and healthcare professionals could use this instrument to measure vaccine intention more broadly; further validity evidence should be generated to affirm its utility with other diseases.

The study found some interesting and not unexpected results that gender and education were correlated with higher confidence/collective responsibility scores. Further, the differences between sites in CA and OH were mostly demographic, but did not translate to differences in 5C domains other than complacency and constraints.

The 5C model addresses confidence (attitudes/beliefs), complacency (risk perceptions), constraints (barriers), calculation (the amount of information searching undergone), and collective responsibility (views on protecting others) [7]. The 5C instrument gathers information on each domain, providing healthcare professionals with areas that impact intention to vaccinate, and allowing for focused conversations related to the respective domain. For example, public trust in vaccines has ebbed and flowed over the years and differs by vaccine often due to confidence in the efficacy and safety of the vaccine [15]. Psychological, social, and political factors all can weigh in on patient confidence [16]. Other research also found “benefits and risks” to be key factors in intention to vaccinate infants [17], receive the COVID-19 vaccine [18], or receive a human papilloma virus (HPV) vaccine [19]. Lower scores in the confidence domain may indicate the need to deliberately schedule conversations with patients about their attitudes and beliefs regarding a vaccine, given that prior work found that vaccination messaging should focus on benefits and risks for influenza and COVID-19 vaccines [15]. With child flu vaccines, which traditionally have shown low intention rates, motivational interviewing (a patient-centered, collaborative conversation) to identify and address attitudes, beliefs, and barriers, has been demonstrated to be effective in decreasing hesitancy and improving vaccine acceptance [20]. Future studies could combine the 5C instrument with interventional research to address the benefits and risks related to COVID-19 and other vaccines with low intentions to vaccinate with patient-centered communication approaches. In addition, while survey results indicate that timing may not impact behavior, an extended study could help to better determine the impact of time (e.g., 2020–early 2021 results with late 2021–early 2022 results pandemic period), particularly since the COVID-19 pandemic has not concluded and different variants of the virus are causing new rises in infection.

The tool also correlated with intention measures for COVID-19, indicating its utility in identifying patients who may be at risk for not accepting a vaccination. It is important to identify intention, as intervention efforts have been found to improve vaccine intention with infant vaccinations [21,22,23], flu and COVID-19 vaccines [11,15], and HPV vaccines [19]. This study also examined intention from two states that are demographically different. There were specific differences by demographics and region, which is consistent with other research [24,25,26]. Capturing the regions with differing intention and acceptance of the COVID-19 vaccine could help inform healthcare officials and policymakers to understand the needs of their citizens, whether it be increasing preventative requirements such as masking and social distancing or requiring vaccination status checks for attendance of public events in enclosed areas to keep their regions safe from infection and spread. Continued challenges with vaccine acceptance remain amidst falling vaccination rates. Ohio, one of the states incorporated in this study, recently experienced a measles outbreak [27,28]. Thus, regional efforts related to vaccinations are promising interventions to promote vaccine intention and acceptance [29,30].

### Limitations

The generalizability of the results may be impacted by the use of a convenience sample based on accessible collaborations in CA and OH, which calls for an expansion of our work in other geographic locations. Another potential limitation was the timing of the questions and adaptability to the evolution of the COVID-19 pandemic. Although the data did not reveal differences across a year in our studies, information about COVID-19 and vaccines in development were mixed and continue to evolve. Hence, the use of certain items in the 5C may need to be evaluated for applicability in each situation [7]. Additionally, some of the items might have benefited from adaptation due to two-part answers required for a response. For example, the item stating “vaccine preventable diseases are not so severe that I should get vaccinated” suggests that the respondent thinks about the severity of the disease then secondly if they consider the vaccination necessary. It may not be clear what their response is relating to for that item specifically, and reveals that a hesitancy survey tailored to the COVID-19 pandemic might have provided more accurate responses.

Not all items included in this study were validated. Thus, results should be interpreted cautiously toward non-5C related items. Further, due to the concurrent flu season, the 5C’s items were left in its general format and did not reference COVID-19 directly in each item: “…the research team is interested in how you personally think about vaccines in general. Please answer how much you disagree or agree with the following statements.” The survey’s latter question about vaccine intention specifies the COVID-19 vaccine. As perceptions towards vaccines have shifted as a result of COVID-19, it may be important to explore the 5C items in relation to specific vaccines. One final limitation is the number of responses gathered from the California sites, which did not reach the minimum desired sample. Further research should explore perceptions in a broader geographic population.

## 5. Conclusions

Given the prevalence of vaccine hesitancy, it is important to understand factors related to vaccine acceptance and intention to vaccinate when having conversations with patients regarding vaccines. The integration of the 5C tool, which continues to demonstrate validity evidence, determined regional differences in vaccine acceptance as well as demographic differences. The 5C tool can be used to examine the impact of key factors on vaccine acceptance. There were differences in states, which indicate that interventions may need to be tailored regionally. Demographics and regions can be targeted for more focused attention regarding COVID-19 vaccination and potential areas to implement more preventative measures by pharmacists. Given that the 5C tool gathers information on different domains of vaccine acceptance, healthcare professionals could utilize these results to improve trust and vaccine confidence in their patient populations; focused conversations concerning any of the respective domains could best address individual concerns and barriers about vaccinations, notably the COVID-19 and flu vaccines.

## Figures and Tables

**Table 1:** Respondent Demographics and Responses to Vaccine Intention Items.

Item	Ohio (*n* = 308)	California (*n* = 47)	Total (*n* = 355)
Gender Identity ^a^, *n* (%)			
Male	143 (46.4)	13 (27.7)	156 (43.9)
Female	165 (53.6)	33 (70.2)	198 (55.8)
Nonbinary or Other	0 (0)	1 (2.1)	1 (0.3)
Age Group ^a^, *n* (%)			
18–29	158 (51.3)	3 (6.4)	161 (45.4)
30–49	81 (26.3)	14 (29.8)	95 (26.8)
50–69	60 (19.5)	27 (57.4)	87 (24.5)
70 or older	7 (2.3)	2 (4.3)	9 (2.5)
Chose not to disclose	2 (0.6)	1 (2.1)	3 (0.8)
Race/Ethnicity ^a^, *n* (%)			
American Indian/Alaskan Native	3 (1.0)	1 (2.1)	4 (1.1)
Asian	9 (2.9)	19 (40.5)	28 (7.9)
Black/African American	3 (1.0)	6 (12.8)	9 (2.5)
Hispanic or Latinx	2 (0.6)	10 (21.3)	12 (3.4)
White	279 (90.6)	7 (14.9)	286 (80.6)
Mixed Race	9 (2.9)	1 (2.1)	10 (2.8)
Highest Educational Qualification ^a^, *n* (%)			
Some High School	8 (2.6)	8 (17.0)	16 (4.5)
High School Diploma	38 (12.3)	12 (25.5)	50 (14.1)
Some College	101 (32.8)	11 (23.4)	112 (31.5)
Associate Degree (e.g., AA, AS)	22 (7.1)	4 (8.5)	26 (7.3)
Bachelor’s Degree (e.g., BA, BS)	81 (26.3)	6 (12.8)	87 (24.5)
Master’s Degree or higher	56 (18.2)	3 (6.4)	59 (16.6)
Chose not to disclose	2 (0.6)	3 (6.4)	5 (1.4)
Yearly Household Income ^a^, *n* (%)			
Less than $20,000	22 (7.1)	15 (31.9)	37 (10.4)
$21,000–$50,000	48 (15.6)	14 (29.8)	62 (17.5)
$51,000–$100,000	86 (27.9)	5 (10.6)	91 (25.6)
$101,000–$150,000	42 (13.6)	0 (0)	42 (11.8)
Greater than $150,000	37 (12.0)	2 (4.3)	39 (11.0)
Chose not to disclose	73 (23.7)	11 (23.4)	84 (23.7)
Respondent Language, *n* (%)			
English	308 (100)	29 (61.7)	337 (94.9)
Spanish	0 (0)	3 (6.4)	3 (0.8)
Chinese	0 (0)	4 (8.5)	4 (1.1)
Vietnamese	0 (0)	11 (23.4)	11 (3.1)
Number of Regularly-Taken Prescription Medications ^a^, *n* (%)			
0	183 (59.4)	13 (27.7)	196 (55.2)
1–2	92 (29.9)	13 (70.2)	105 (29.6)
3–4	26 (8.4)	16 (34.0)	42 (11.8)
6 or more	7 (2.3)	5 (10.6)	12 (3.4)
Not reported	0 (0)	0 (0)	153 (43.1)
“I got the flu vaccine last year” (2019–2020) ^a^, *n* (%)			
Yes	124 (40.3)	32 (68.1)	156 (43.9)
No	167 (54.2)	14 (29.8)	181 (51.0)
I don’t remember	17 (5.5)	1 (2.1)	18 (5.1)
“I plan to get (or have already received) the flu shot this year (2020–2021)” **, *n* (%)			
Yes, I plan to get the flu shot	86 (27.9)	26 (55.3)	112 (31.5)
Yes, I have already received the flu shot	69 (22.4)	12 (25.5)	81 (22.8)
No	110 (35.7)	5 (10.6)	115 (32.4)
Unsure	43 (14.0)	2 (4.3)	45 (12.7)
I will get (or have already received the flu vaccine this year because… ^a,b^, *n* (%)			
It is mandatory for my work	34	7	41
I believe it will help	113	27	140
I know how it helps	70	17	87
I think it is important to protect others	84	19	103
I may not take the flu vaccine this year because… ^b^, *n* (%)			
It will give me the flu	13	4	17
I do not believe it helps	85	1	86
I do not know how it will help	62	3	65
I won’t be interacting with others	9	1	10
Has anyone close to you been directly impacted by the COVID-19 pandemic? ^a^, *n* (%)			
Yes	238 (77.3)	19 (40.4)	257 (72.4)
No	56 (18.2)	22 (46.8)	78 (22.0)
I don’t know	14 (4.5)	6 (12.8)	20 (5.6)
If the COVID-19 vaccine becomes available, select ONE of the following statements that best fits your opinion ^a^, *n* (%)… I will:			
…get the COVID vaccine immediately when it is available	72 (23.4)	25 (53.2)	97 (27.3)
…get the COVID vaccine only when it has been out for a few months	155 (50.3)	20 (42.6)	175 (49.3)
…not get the COVID vaccine because I don’t want to have side effects	53 (17.2)	1 (2.1)	54 (15.2)
…not get the COVID vaccine because I don’t need it	28 (9.1)	1 (2.1)	29 (8.2)

^a^ Pearson Chi-Square indicated a significant difference between OH and CA (*p* = 0.000–0.003). ^b^ Select all that apply; thus, percentages were not calculated for items with multiple responses available. ** Missing 2 responses due to Qualtrics error.

**Table 2:** 13-item 5C with Forced Four Factor, Varimax Rotation.

	Component			
	Confidence/ Collective Responsibility	Constraints	Calculation	Complacency
Vaccinations are effective.	0.703	0.211	0.104	0.049
I am completely confident that vaccines are safe.	0.785	0.102	0.246	0.055
Regarding the provisions of vaccinations, I am confident that public authorities decide in the best interest of the community.	0.648	−0.003	0.192	0.181
My immune system is so strong, it also protects me against diseases.	0.024	0.034	−0.003	0.929
Vaccine-preventable diseases are not so severe that I should get vaccinated. [R]	0.417	0.370	0.175	0.265
For me, it is inconvenient to receive vaccinations. [R]	0.176	0.675	0.036	0.163
Visiting the doctor’s makes me feel uncomfortable, this keeps me from getting vaccinated. [R]	0.186	0.841	−0.020	−0.104
Everyday stress prevents me from getting vaccinated. [R]	0.152	0.856	−0.025	−0.106
For each and every vaccination, I closely consider whether it is useful for me. [R]	0.143	0.143	0.774	0.033
It is important for me to fully understand the topic of vaccination, before I get vaccinated. [R]	0.074	−0.019	0.835	−0.037
When I think about getting vaccinated, I weigh benefits and risks to make the best decision possible. [R]	0.075	−0.093	0.838	0.024
I get vaccinated because I can also protect people with a weaker immune system.	0.735	0.177	−0.084	0.134
Vaccination is a collective action to prevent the spread of diseases.	0.722	0.259	−0.031	0.118

Extraction Method: Principal Component Analysis. Rotation Method: Varimax with Kaiser Normalization. Rotation converged in 5 iterations. [R] = recoded per 5C protocol to match positive connotation of acceptance.

**Table 3:** Mean Scores from 13-item 5C Domain, Full Questionnaire, and Total Minimum to Maximum *.

	Ohio (*n* = 308)	California (*n* = 47)	Total (*n* = 355)
Confidence/Collective Responsibility Domain			
Mean score (SD)	3.04 (0.83)	3.19 (0.50)	3.06 (0.50)
Total score (SD)	15.22 (2.48)	15.98 (2.52)	15.32 (2.50)
Total score, min-max (possible 5–20)	9–20	10–20	9–20
Complacency Domain *			
Mean score (SD)	2.82 (0.32)	2.53 (0.57)	2.79 (0.50)
Total score (SD)	5.64 (0.97)	5.06 (1.13)	5.57 (1.01)
Total score, min-max (possible 2–8)	3–8	3–7	3–8
Constraints Domain *			
Mean score (SD)	3.28 (0.49)	3.06 (0.64)	3.25 (0.52)
Total score (SD)	9.84 (1.47)	9.19 (1.93)	9.76 (1.55)
Total score, min-max (possible 3–12)	6+–12	4–12	4–12
Calculation Domain			
Mean score (SD)	2.82 (0.62)	2.89 (0.49)	2.83 (0.61)
Total score (SD)	8.45 (1.87)	8.68 (1.48)	8.48 (1.82)
Total score, min-max (possible 3–12)	3–12	6–12	3–12
13-item 5C Total			
Total score (SD)	39.16 (3.90)	38.91 (4.16)	39.13 (3.93)
Total score, min-max (possible 13–52)	30–51	31–47	30–51

* Independent *t*-test showed significant differences (2-tailed) between groups (*p* = 0.000–0.030). Levene’s Test for Equality of Variances showed no significance between groups in any domain or the total 5C score.

**Table 4:** Associations between 5C Domains and Demographic Characteristics determined by ANOVA (*n* = 355).

	Confidence/ Collective Responsibility Domain	Complacency Domain	Constraints Domain	Calculation Domain	13-Item 5C Total
Gender Identity, mean (SD)					
Male	3.03 (0.49)	2.82 (0.49)	3.21 (0.49)	2.76 (0.55)	38.66 (3.61)
Female	3.10 (0.51)	2.76 (0.52)	3.29 (0.54)	2.88 (0.64)	39.53 (4.13)
Nonbinary or Other *		2.00	3.00	2.67	33.00
Significant associations, *p*-value	---	---	---	---	0.035 **
Age Group, mean (SD)					
18–29	3.05 (0.50)	2.84 (0.51)	3.22 (0.51)	2.76 (0.67)	38.88 (4.03)
30–49	3.06 (0.50)	2.79 (0.48)	3.26 (0.50)	2.92 (0.51)	39.43 (3.47)
50–69	3.09 (0.51)	2.68 (0.50)	3.30 (0.56)	2.84 (0.59)	39.24 (4.31)
70 or older	3.22 (0.46)	2.83 (0.61)	3.33 (0.55)	2.85 (0.63)	40.33 (3.08)
Chose not to disclose	2.80 (0.35)	2.33 (0.58)	3.11 (0.19)	2.78 (0.19)	36.33 (3.06)
Significant associations, *p*-value	---	---	---	---	---
Race/Ethnicity, mean (SD)					
American Indian/Alaskan Native	2.40 (0.16)	2.25 (0.29) ^a^	3.08 (0.17)	2.92 (0.17)	34.50 (1.29)
Asian	3.16 (0.27)	2.50 (0.43)	3.08 (0.58)	2.76 (0.61)	38.36 (3.50)
Black/African American	2.96 (0.65)	2.89 (0.55)	3.19 (0.56)	3.07 (0.46)	39.33 (4.30)
Hispanic or Latinx	3.30 (0.71)	2.50 (0.60) ^a^	3.17 (0.81)	3.03 (0.41)	40.08 (5.12)
White	3.06 (0.50)	2.83 (0.50)	3.28 (0.49)	2.82 (0.60)	39.28 (3.94)
Mixed Race	3.10 (0.61)	2.65 (0.53)	3.13 (0.61)	2.50 (1.02)	37.70 (2.98)
Significant associations, *p*-value	---	0.001	---	---	---
Highest Educational Qualification, mean (SD)					
Some High School	3.04 (0.39)	2.44 (0.48) ^a,b^	3.13 (0.58)	2.69 (0.59)	37.50 (3.12) ^a^
High School Diploma	3.01 (0.46)	2.64 (0.48) ^c^	3.15 (0.54) ^a^	2.84 (0.50)	38.32 (4.13) ^b^
Some College	3.02 (0.48) ^a^	2.77 (0.48)	3.22 (0.49) ^b^	2.89 (0.65)	38.96 (4.02) ^c^
Associate Degree (e.g., AA, AS)	2.98 (0.54)	2.73 (0.57)	3.00 (0.59) ^b^	2.97 (0.41)	38.27 (4.41)
Bachelor’s Degree (e.g., BA, BS)	3.07 (0.53)	2.90 (0.52) ^a^	3.31 (0.47)	2.76 (0.63)	39.38 (3.57)
Master’s Degree or higher	3.26 (0.48) ^a^	2.92 (0.45) ^b,c^	3.49 (0.47) ^a^	2.75 (0.66)	40.90 (3.58) ^a,b,c^
Chose not to disclose	2.60 (0.51)	2.30 (0.27)	2.80 (0.18)	3.13 (0.30)	35.40 (2.30)
Significant associations, *p*-value	0.012	0.000	0.000	---	0.001
Number of Regularly-Taken Prescription Medications, mean (SD)					
0	3.01 (0.49) ^a^	2.79 (0.49)	3.22 (0.50)	2.79 (0.60)	38.65 (3.77)
1–2	3.09 (0.51)	2.83 (0.51)	3.29 (0.53)	2.86 (0.64)	39.59 (4.11)
3–4	3.22 (0.46) ^a^	2.68 (0.55)	3.21 (0.56)	2.88 (0.56)	39.73 (3.93)
6 or more	3.20 (0.52)	2.58 (0.63)	3.58 (0.47)	2.97 (0.50)	40.83 (4.30)
Significant associations, *p*-value	0.039	---	---	---	---
“I got the flu vaccine last year” (2019–2020), mean (SD)					
Yes	3.22 (0.49) ^a,b^	2.86 (0.54) ^a^	3.38 (0.53) ^a,b^	2.75 (0.64) ^a^	40.21 (4.07) ^a,b^
No	2.94 (0.48) ^a^	2.71 (0.48) ^a^	3.17 (0.48) ^a^	2.92 (0.57) ^a,b^	38.42 (3.63) ^a^
I don’t remember	2.93 (0.39) ^b^	2.83 (0.30)	2.96 (0.47) ^b^	2.57 (0.51) ^b^	36.94 (3.26) ^b^
Significant associations, *p*-value	0.000	0.020	0.000	0.005	0.000
“I plan to get (or have already received) the flu shot this year (2020–2021)”, mean (SD)					
Yes, I plan to get the flu shot	3.25 (0.44) ^a,b^	2.85 (0.54)	3.35 (0.59) ^a,b^	2.70 (0.55) ^a^	40.08 (4.11) ^a,b^
No	2.83 (0.49) ^a,c^	2.69 (0.49) ^a^	3.13 (0.46) ^a,c^	2.98 (0.58) ^a^	37.84 (3.64) ^a,c^
Yes, I have already received the flu shot	3.23 (0.46) ^c,d^	2.90 (0.50) ^a^	3.42 (0.46) ^c,d^	2.77 (0.73)	40.48 (3.77) ^c,d^
Unsure	2.91 (0.37) ^b,d^	2.66 (0.40)	3.04 (0.42) ^b,d^	2.87 (0.50)	37.62 (3.08) ^b,d^
Significant associations, *p*-value	0.000	0.006	0.000	0.003	0.000
Has anyone close to you been directly impacted by the COVID-19 pandemic? mean (SD)					
Yes	3.06 (0.50)	2.83 (0.50) ^a^	3.27 (0.51)	2.85 (0.64)	39.32 (4.04)
No	3.11 (0.52)	2.67 (0.51) ^a^	3.24 (0.52)	2.80 (0.50)	38.99 (3.67)
I don’t know	2.96 (0.40)	2.63 (0.48)	3.07 (0.54)	2.67 (0.48)	37.25 (3.16)
Significant associations, *p*-value	---	0.015	---	---	---
If the COVID-19 vaccine becomes available, select ONE of the following statements that best fits your opinion, mean (SD)I will:					
…get the COVID vaccine immediately when it is available	3.43 (0.43) ^a,b,c^	2.85 (0.60) ^a^	3.47 (0.51) ^a,b,c^	2.71 (0.68)^a^	41.35 (3.63) ^a,b,c^
…get the COVID vaccine only when it has been out for a few months	3.06 (0.40) ^a,d,e^	2.82 (0.46) ^b^	3.21 (0.50) ^a^	2.83 (0.60)	39.05 (3.60) ^a,d,e^
…not get the COVID vaccine because I don’t want to have side effects	2.65 (0.43) ^b,d^	2.56 (0.41) ^a,b^	3.13 (0.47) ^b^	3.02 (0.47) ^a^	36.81 (3.34) ^b,d^
…not get the COVID vaccine because I don’t need it	2.63 (0.43) ^c,e^	2.76 (0.51)	3.05 (0.52) ^c^	2.89 (0.53)	36.48 (3.79) ^c,e^
Significant associations, *p*-value	0.000	0.003	0.000	0.024	0.000

* No SD could be calculated due to single value point. ** No Tukey’s could be calculated due to only 1 nonbinary respondent. ^a–e^ Significant differences between groups, *p* < 0.05, Tukey’s.

## Data Availability

Please contact Anandi Law for any data.
